# No effect of cancer-associated SNP rs6983267 in the 8q24 region on co-expression of *MYC *and *TCF7L2 *in normal colon tissue

**DOI:** 10.1186/1476-4598-8-96

**Published:** 2009-11-06

**Authors:** Ludmila Prokunina-Olsson, Jennifer L Hall

**Affiliations:** 1 Laboratory of Translational Genomics, Division of Cancer Epidemiology and Genetics, National Cancer Institute, National Institutes of Health, Bethesda, 20892, MD, USA; 2 Lillehei Heart Institute, Department of Medicine, University of Minnesota, Minneapolis, 55455, MN, USA

## Abstract

A single nucleotide polymorphism (SNP) rs6983267, located within the 8q24 region, is strongly associated with risk of colorectal and prostate cancer. It has been suggested that the mechanism of this association is related to differential interaction of TCF7L2 protein (previously known as TCF-4) with alleles of rs6983267, influencing the expression of a well-known oncogene, *MYC*, located 335 Kb telomeric. Here, we tested the correlation between mRNA expression of *MYC *and several alternatively spliced forms of *TCF7L2 *in 117 non-cancer colon samples. We observed a strong correlation (r = 0.60, p < 10^-6^) between expression of *MYC *and a unique splicing form of *TCF7L2*. The level of *MYC *expression in these samples was associated with expression of some *TCF7L2 *splicing forms but not with genotypes of rs6983267, or interaction of rs6983267 with *TCF7L2 *expression. These findings suggest that some splicing forms of *TCF7L2 *may be functionally important for regulation of *MYC *expression in colon tissue but this regulation is not directly dependent on rs6983267.

## Findings

Recent genome-wide association studies (GWAS) have identified a single nucleotide polymorphism (SNP) rs6983267 within the 8q24 region associated with increased susceptibility to colorectal and prostate cancer [1-6]. Follow-up association studies have suggested that the same variant may also increase the risk for cancers of the kidney, thyroid and larynx [7,8]. The location of rs6983267 in the intergenic region 335 Kb upstream from the *MYC *gene, a well-known oncogene [9], generated a hypothesis that this SNP might be involved in a long-distance regulation of *MYC *expression. Located in a region with significant evolutionary conservation and enhancer potential [10-12], the SNP was predicted to affect a binding site for TCF7L2 [10,11], a key transcription factor in the WNT pathway. The risk allele G of rs6983267 was found to have a slightly stronger affinity to TCF7L2 in binding assays compared to the non-risk allele T, and stronger regulatory activity in luciferase reporter assays [10,11]. An analysis of long-range interactions showed that the region containing rs6983267 might be in physical proximity with *MYC *region [10]. These findings suggested that rs6983267 might be located within an enhancer element that interacts with TCF7L2 and regulates *MYC *expression [10,11]. *MYC *is a target gene of TCF7L2 [13-15] and its expression is regulated through two TCF7L2 binding sites within the *MYC *promoter [13]. No association has been found between rs6983267 and the mRNA expression of *MYC *in lymphoblastoid cell lines [11,16], normal and tumor colon samples [10,11,17-20], or with MYC immunostaining in colon tumors [5].

Previously, we performed a detailed study of *TCF7L2 *expression in several types of human tissue, including colon where we measured the expression of multiple assays targeting the majority of known splicing forms of *TCF7L2 *[21,22]. In the current study we sought to determine, whether the expression of *TCF7L2 *splicing forms we identified in non-cancer colon samples correlated with *MYC *expression and whether this expression was dependent on alleles of rs6983267 or interaction of rs6983267 with *TCF7L2 *expression.

**Figure 1:**
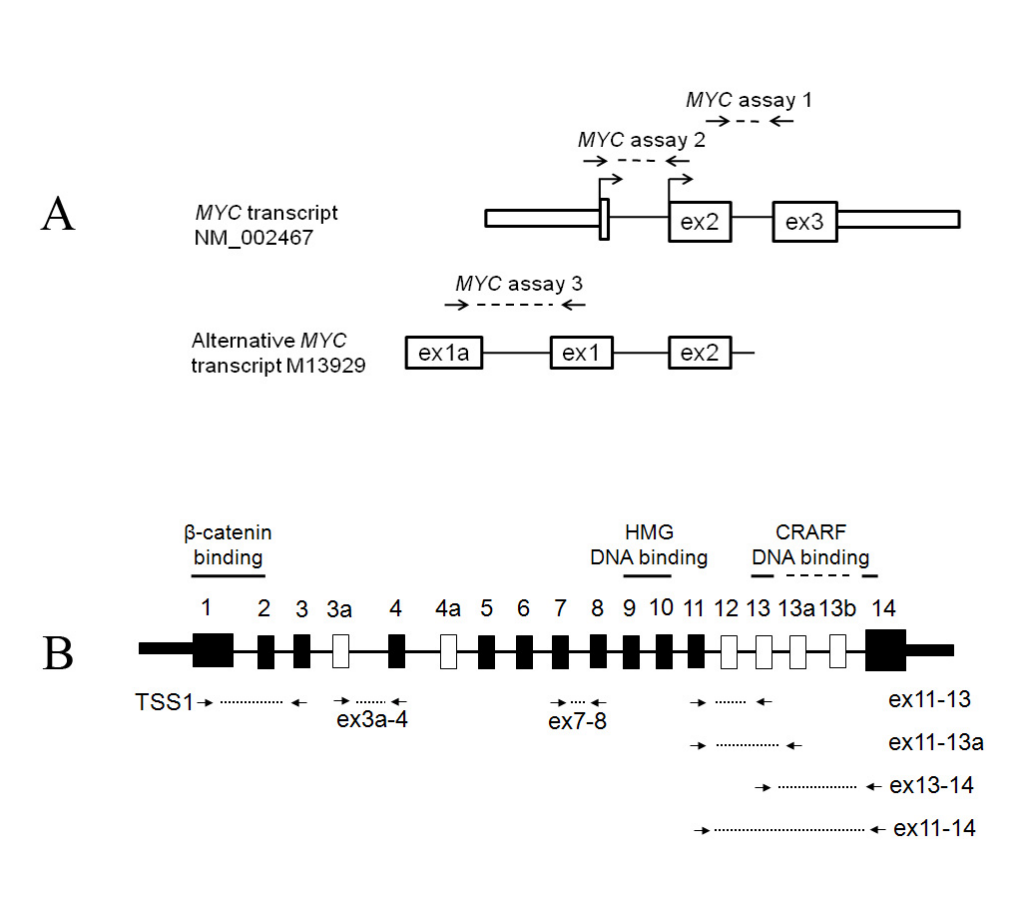
**Location of *MYC *and *TCF7L2 *expression assays**. **A**. *MYC *exons and 5' and 3'untranslated regions (UTRs) are marked by rectangles and two translation starts are marked by vertical lines and arrows. *MYC *assay1 is located over the junction of exons 2 and 3, and *MYC *assay 2 is located over the junction of exons 1 and 2. *MYC *assay 3 targets alternative transcript with an upstream exon. **B**. Constitutive exons of *TCF7L2 *are represented as black rectangles and alternative exons as white rectangles. Protein domains are indicated above corresponding exons: β-catenin binding domain is encoded by exons 1 and 2, high mobility group (HMG) DNA-binding domain is encoded by exons 9 and 10 and the CRARF DNA-binding domain is encoded by exons 13 and 14. Location of expression assays is indicated under corresponding exons and arrows show primer positions. The specificity of detection of particular splicing forms is achieved by probes located over exon junctions.

We investigated non-cancer colon samples on the assumption that the effect of a germline genetic variation might be more easily detectable in conditions not affected by the effects of cancer or its treatment. The samples and the methods are described in Additional file 1. The mRNA expression of *MYC *was detected by sensitive quantitative reverse-transcriptase PCR (qRT-PCR) and 3 expression assays (Figure 1A.) The expression of *MYC *assays 1 and 2, corresponding to exons 2-3, and 1-2, respectively (RefSeq transcript NM_002467), was highly correlated (r = 0.95). *MYC *assay 3 targeted an alternative transcript initiated from a promoter P0 (GenBank accession number M13929) [23], however, expression of this transcript was very low (at a level of at least 100 times lower than of assays 1 and 2) and was not studied further. Expression of *TCF7L2 *was measured with 7 assays previously described (Figure 1B, Additional file 2) [21,22].

**Figure 2:**
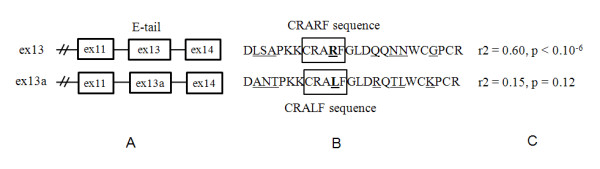
**Detailed structure of C-terminal part of *TCF7L2 *gene**. **A**. Combinations of exons 11, 13, 13a and 14 of *TCF7L2 *encoding proteins with long reading frames (E-tail). **B**. A form with alternative exon 13 encodes a protein sequence with CRARF motif; a form with alternative exons 13a encodes a protein sequence with CRALF motif, differences in amino acids in proteins encoded by exons 13 and 13a are underlined, CRARF and CRALF motifs are boxed. **C**. Expression of a splicing form with exon13 correlates with *MYC *expression (r = 0.60, p < 10^-6^), while expression of a splicing form with exon 13a does not correlate with *MYC *expression (r = 0.15, p = 0.12).

The strongest correlation between *MYC *and *TCF7L2 *expression was observed for assay "ex13-14" of *TCF7L2 *(r = 0.57- 0.60, p < 10^-6^), followed by assay "ex11-13" (r = 0.52-0.54, p < 10^-6^). The weakest correlation was detected for assay "ex11-13a" (r = 0.10 - 0.15, p = 0.12 - 0.28) (Table 1). These assays detect alternative splicing forms that include combinations of exons 11-13-14 and 11-13a-14 in the C-terminal end of the *TCF7L2 *transcripts (GenBank accession numbers FJ010174 and FJ010167). Both protein isoforms encoded by these splicing forms have long C-terminal reading frames (E-tails) with binding sites for the C-terminal binding protein (CtBP) involved in post-translational regulation of TCF7L2 expression [21,22]. Protein fragments encoded by the alternative exons 13 and 13a share 68% identity (17 amino acids of 25, Figure 2). The form with exons 11-13-14 encodes a 30-amino-acid highly conserved motif with a CRARF signature protein sequence, while in the form with exons 11-13a-14 this sequence is changed to CRALF (Figure 2). The CRARF protein sequence is also found in another member of TCF/LEF family of transcription factors, TCF-7 (former TCF-1) and in the ancestral drosophila TCF/pangolin protein [24]. The CRARF sequence serves as an additional DNA-binding domain and a strong transactivator of the WNT pathway [24,25]. The CRARF-form of TCF7L2 was shown to interact with two TCF7L2 binding sites within *MYC *promoter, TBE1 at -1156 bp and TBE2 at -589 bp upstream the first translation start site [13,24]. Therefore, we suggest that while both the CRARF and CRALF forms of TCF7L2 contain E-tails, only the CRARF form regulates *MYC *expression in the colon. A splicing form detected by assay "ex11-14" is the most common splicing form of *TCF7L2 *in all human tissues [21,22]. This splicing combination utilizes an alternative stop codon in the beginning of exon 14 resulting in a protein without the CtBP-binding domain. A somatic frameshift mutation in a polyA stretch within exon 14 found in colorectal cancer cell lines results in similar outcome - termination of protein by an alternative stop codon in the beginning of exon 14 [26,27]. We found a moderate correlation between assay "ex11-14" of *TCF7L2 *and *MYC *expression (Table 1). Next, we evaluated the levels of mRNA *MYC *expression in colon tissue in relation to genotypes of rs6983267. We observed significant effect of age, of several *TCF7L2 *splicing forms but no effect of rs6983267 alone or in interaction with *TCF7L2 *(Table 2).

**Table 1:** Correlation between expression of *MYC *and *TCF7L2 *in colon samples

***TCF7L2*** **assays**	***MYC *assay 1**		***MYC *assay 2**	
	
	r*	**p-value#**	r*	**p-value#**
*TCF7L2 *TSS1	0.23	0.014	0.21	0.023
*TCF7L2 *ex3a-4	0.17	0.070	0.15	0.12
*TCF7L2 *ex7-8	0.38	2.4 × 10^-5^	0.34	2.1 × 10^-4^
*TCF7L2 *ex11-13a	0.15	0.12	0.10	0.28
*TCF7L2 *ex11-14	0.40	1.2 × 10^-5^	0.34	2.5 × 10^-4^
*TCF7L2 *ex11-13	0.54	<10^-6^	0.52	<10^-6^
*TCF7L2 *ex13-14	0.60	<10^-6^	0.57	<10^-6^

*Spearman correlation coefficient and # two-sided p-values for *MYC *and *TCF7L2 *expression in 117 colon samples, p-values are not adjusted for multiple tests

**Table 2:** Association of rs6983267 with *MYC *expression in colon tissue

**factors**	*MYC *assay 1, p-value *	*MYC *assay 2, p-value *
^a^rs6983267	0.85	0.91

^b^age	0.040	0.043
^b^rs6983267	0.83	0.84

age	0.13	0.13
Rs6983267	0.85	1.00
*TCF7L2 *TSS1	0.054	0.076
rs6983267*TCF7L2 TSS1	0.91	0.91

age	0.069	0.062
Rs6983267	1.00	0.97
*TCF7L2 *ex3a-4	0.36	0.52
rs6983267*TCF7L2 ex3a-4	0.93	0.77

Age	0.31	0.39
Rs6983267	0.75	0.28
*TCF7L2 *ex7-8	0.0039	0.021
rs6983267*TCF7L2 ex7-8	0.79	0.25

age	0.045	0.037
Rs6983267	0.95	0.90
*TCF7L2 *ex11-13a	0.31	0.50
rs6983267*TCF7L2 ex11-13a	0.97	0.89

age	0.38	0.37
Rs6983267	0.70	0.61
*TCF7L2 *ex11-14	0.0008	0.013
rs6983267*TCF7L2 ex11-14	0.57	0.50

age	0.13	0.19
Rs6983267	0.22	0.41
*TCF7L2 *ex11-13	<0.0001	<0.0001
rs6983267*TCF7L2 ex11-13	0.29	0.48

age	0.25	0.27
Rs6983267	0.28	0.30
*TCF7L2 *ex13-14	<0.0001	<0.0001
rs6983267*TCF7L2 ex13-14	0.35	0.35

*p-values for univariate regression analysis including 0, 1 and 2 alleles of rs6983267 and expression of *MYC *assays 1 and 2 in the presence of covariates -- age and level of expression of TCF7L2 assays or interaction between rs6983267 and *TCF7L2 *expression; p-values are not adjusted for multiple tests; ^a ^genotypes were available for 96 samples; ^b ^age and genotypes were available for 79 samples.

Our results show a strong role of *TCF7L2 *in regulation of *MYC *expression in colon, but not through rs6983267. Both *TCF7L2 *and *MYC *genes are expressed in colon and are important for maintaining proliferation of intestinal epithelium [28,29] (Additional file 3). Inactivation of the adenomatous polyposis coli (*APC*) tumor suppressor gene leads to formation of β-catenin/TCF7L2 complexes, constitutive activation of the WNT pathway and eventually colorectal cancer. Rare point mutations within *TCF7L2 *are also found in colorectal cancers [30]. The proliferative effect of TCF7L2 is achieved through its transcriptional regulation of several target genes such as *MYC *and *CCND1 *(*Cyclin D1)*[31]. Our results suggest that expression of *MYC *in colon tissue is most likely regulated by a splicing form of *TCF7L2 *encoding a protein with a potent transactivation CRARF-domain. However, we did not find any evidence for an effect of rs6983267 on *TCF7L2 *regulation of *MYC *expression. Of the family of TCF/LEF transcription factors, *TCF7L2 *has the highest expression in colon, but other members of this family may also be involved. Inactivation of TCF-7 (former TCF-1) leads to development of intestinal polyps [32]. Expression of LEF1 is found in tumors but not in normal colon tissue [33]. Each of these proteins can recognize the same TCF/LEF consensus binding site and, therefore, might bind alleles of rs6983267. High degree of similarity between TCF/LEF proteins may lead to cross-reactivity in chromatin immunoprecipitation (ChIP) assays. Thus, other TCF/LEF factors should also be examined for their effect on regulation of *MYC *expression.

In conclusion, SNP rs6983267 within the 8q24 region has been established as one of the strongest genetic risk factors for development of at least two types of cancer. Identification of functional mechanisms of this association is the highest priority of cancer genetics and would mean a significant step forward towards understanding of cancer pathogenesis and development of better diagnostic and therapeutic approaches. Our results provide new insights into the regulation of *MYC *expression by TCF7L2. However, further studies are needed to investigate alternative molecular mechanisms that can explain the association between rs6983267 and cancer risk.

## Competing interests

The authors declare that they have no competing interests.

## Authors' contributions

LPO designed and performed the study and wrote manuscript, JLH provided samples and wrote manuscript. Both authors read and approved the final manuscript.

## Supplementary Material

Additional file 1 **Materials and methods**. The data provided represent the materials, methods and statistical analysis used to study mRNA coexpression of *MYC *and *TCF7L2*. Click here for file

Additional file 2 **Expression assays used in this study**. primers, probes and TaqMan assay IDs. Click here for file

Additional file 3 **Protein expression of MYC and TCF7L2 in normal human colon**. A. Expression of MYC in normal human colon; B. Expression of TCF7L2 in normal human colon. Both proteins show glandular staining in colon epithelium. Images are courtesy of Protein Atlas Click here for file
