# Supplementary material for: No effect of cancer-associated SNP rs6983267 in the 8q24 region on co-expression of MYC and TCF7L2 in normal colon tissue
Source: Mol Cancer. 2009 Nov 6;8:96. doi: 10.1186/1476-4598-8-96 (PMC2777153; doi:10.1186/1476-4598-8-96)
Supplement: Additional file 1 — Materials and methods. The data provided represent the materials, methods and statistical analysis used to study mRNA coexpression of MYC and TCF7L2. [file 1476-4598-8-96-S1.doc]

**Additional file 1.**

**Materials and methods**

De-identified samples of fresh-frozen colon epithelial tissue were provided by the tissue procurement facility at the University of Minnesota. The samples were surgery biopsies and represented non-cancer tissue according to pathology reports. The study was approved by the IRB at the University of Minnesota. DNA from tissue samples was prepared with DNAeasy (Qiagen) and RNA was prepared with RNAeasy (Qiagen). cDNA for all samples was prepared from 0.5 ug of DNAse-treated total RNA with SuperScript III kit and random hexamers (Invitrogen) and was diluted 20-fold with water. The qRT-PCR reactions were performed in duplicates in a 5ul reaction volume with 2xGene Expression buffer (Applied Biosystems) and an equivalent of a 2 ng of total RNA per reaction on a 7900 Real-Time PCR System (Applied Biosystems). *TCF7L2* expression assays were previously described (1). Based on previous study, the assays detecting transcripts of *TCF7L2* with very low level of expression in colon tissue were not used (1). Information about all expression assays and PCR conditions is provided in Additional file 2. Genotyping of DNA samples has been performed on a 7900 Real-Time PCR System (Applied Biosystems) with 5 ng of DNA in a 5 ul reaction volume with 2xGenotyping Master Mix and an allelic discrimination assay for rs6983267 (C_29086771_20, Applied Biosystems).

**Statistical analysis**

For each sample and assay, expression values for technical duplicates were averaged and the values were accepted if the standard deviations did not exceed 0.5 Ct values (Ct value is a PCR cycle at which the signal is detected). Expression of all *TCF7L2* and *MYC* assays was normalized to the expression of endogenous control *B2M*. The Log 2 transformed normalized expression values of *MYC* and *TCF7L2* assays passed the normality test. The correlation between *MYC* and *TCF7L2* assays was tested with Pearson correlation coefficient and the significance was evaluated with p-values for two-sided tests. We evaluated expression of MYC assays according to model MYC = TCF7L2 + age+SNP + SNP*TCF7L2, where age and expression of *TCF7L2* assays was included as covariates (one assay at the time) and genotypes of rs6983537 were coded as 0, 1 and 2 dependent on counts of risk alleles. The p-values were not adjusted for multiple tests. All the analyses were performed with SPSS 16.0 software (SPSS Inc. Chicago, Illinois) and SAS/STAT system version 9.2 (SAS Institute Inc.)

1 Prokunina-Olsson, L., Welch, C., Hansson, O., Adhikari, N., Scott, L.J., Usher, N., Tong, M., Sprau, A., Swift, A., Bonnycastle, L.L. *et al.* (2009) Tissue-specific alternative splicing of TCF7L2. *Hum Mol Genet*, **18**, 3795-3804.
