# Supplementary material for: No effect of cancer-associated SNP rs6983267 in the 8q24 region on co-expression of MYC and TCF7L2 in normal colon tissue
Source: Mol Cancer. 2009 Nov 6;8:96. doi: 10.1186/1476-4598-8-96 (PMC2777153; doi:10.1186/1476-4598-8-96)
Supplement: Additional file 2 — Expression assays used in this study. primers, probes and TaqMan assay IDs. [file 1476-4598-8-96-S2.doc]

Additional file 2.

| **Expression assays used in this study** | | |
| --- | --- | --- |
| Expression assays, Type | Primers, probes or commercial assays from Applied Biosystems | Amplicon  size, bp |
| TCF7L2,TSS1  SYBR Green | P1ex1F: GGTGGAGGGGATGACCTA  Ex3R: GGGATCATGATGAAGGGGTA | 307 bp |
| TCF7L2, ex3a-ex4  SYBR Green | Ex3aF: ACTCTGCGTACAAAACGATTGA  Ex4R: AGCAGTGGCCATTTCATCTG | 66 bp |
| TCF7L2, ex7-8,  TaqMan | Hs00181036_m1 |  |
| TCF7L2,  ex11-13, TaqMan | Ex11F: GAAGAAGAGGAAAAGGGACAAGCA  Ex13R: GCGCTCGGCATTTCTTAGGA  FAM-probe: CAGGTCATTGGTCTCTCC | 70 bp |
| TCF7L2,  ex11-13a, TaqMan | Ex11F: GAAGAAGAGGAAAAGGGACAAGCA  Ex13aR: CGGTCAAGCCCGAACAGT  FAM-probe: ACCAATGATGCAAATACT | 86 bp |
| TCF7L2,  ex11-14, TaqMan | Ex11F: CGCGGGATAACTATGGAAAGAAGAA  Ex14R: CTTGTATGTAGCGAACGCACTTTT  FAM-probe: TTTTCTCATTGGTCTCTCCCGGCTG | 94 bp |
| TCF7L2,  ex13-14, TaqMan | Ex13F: GCTTTGGCCTTGATCAACAGAATAA  Ex14R: CTTGTATGTAGCGAACGCACTTTT  FAM-probe: TCTCCTGCAAGGGCC | 74 bp |
| MYC, assay 1  TaqMan | HS00153408_m1 |  |
| MYC, assay 2  TaqMan | HS00905030_m1 |  |
| MYC, assay 3  TaqMan | HS01562521_m1 |  |
| Beta-2-microglobulin (B2M) TaqMan | Hs00187842_m1 |  |

Conditions: SYBR Green assays were run with 2x Power SYBR master mix (Applied Biosystems) and TaqMan assays were run with 2x Gene Expression Mix (Applied Biosystems) with standard conditions (95C- 10 min, and 40 cycles of 95C-15 sec, 60C-1 min).
