# Supplementary material for: No effect of cancer-associated SNP rs6983267 in the 8q24 region on co-expression of MYC and TCF7L2 in normal colon tissue
Source: Mol Cancer. 2009 Nov 6;8:96. doi: 10.1186/1476-4598-8-96 (PMC2777153; doi:10.1186/1476-4598-8-96)
Supplement: Additional file 3 — Protein expression of MYC and TCF7L2 in normal human colon. A. Expression of MYC in normal human colon; B. Expression of TCF7L2 in normal human colon. Both proteins show glandular staining in colon epithelium. Images are courtesy of Protein Atlas [file 1476-4598-8-96-S3.doc]

**Additional file 3**

A. Expression of MYC in normal human colon; B. Expression of TCF7L2 in normal human colon. Both proteins show glandular staining in colon epithelium. Images are courtesy of Protein Atlas [www.proteinatlas.org](http://www.proteinatlas.org/) (1).

A.
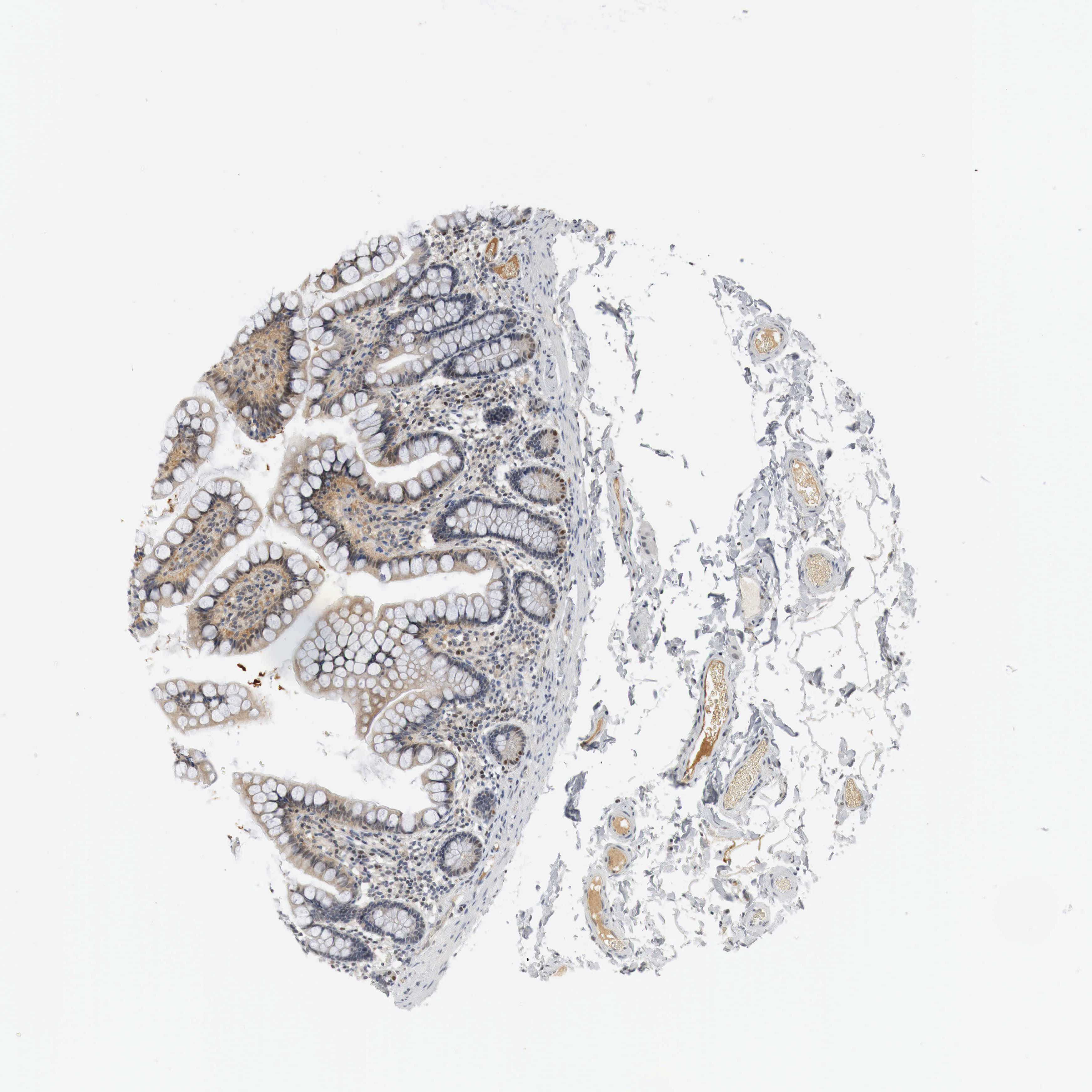


B.
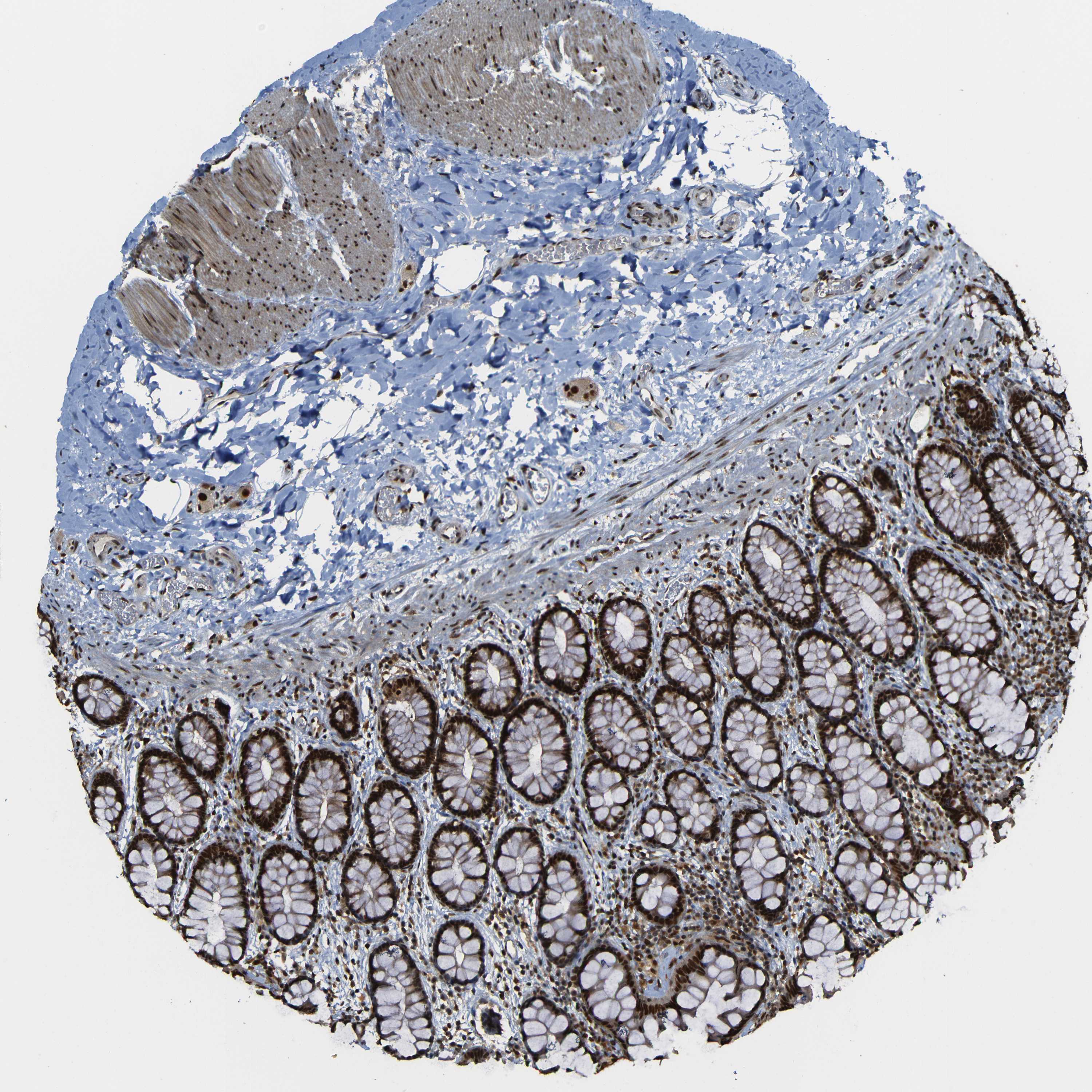


1 Uhlen, M., Bjorling, E., Agaton, C., Szigyarto, C.A., Amini, B., Andersen, E., Andersson, A.C., Angelidou, P., Asplund, A., Asplund, C. *et al.* (2005) A human protein atlas for normal and cancer tissues based on antibody proteomics. *Mol Cell Proteomics*, **4**, 1920-1932.
